# Deeper Pathways for Recruitment Efforts: Identifying Opportunities for Undergraduate and Medical Student Involvement in Infectious Diseases

**DOI:** 10.1093/ofid/ofad439

**Published:** 2023-08-23

**Authors:** Divyam Goel, Michelle T Tin, Krishna C Hariprasad, Diya S Garg, Arnel Besic, Tilly A Dillon, Zoe R Masson, Lauren A Goralsky, Julia A Goralsky, Molly K Barron, Jasmine A Saji, Wendy L Hobson, Trahern W Jones

**Affiliations:** College of Science, University of Utah, Salt Lake City, Utah, USA; College of Science, University of Utah, Salt Lake City, Utah, USA; College of Natural Sciences, Department of Molecular Biosciences, University of Texas at Austin, Austin, Texas, USA; College of Science, University of Utah, Salt Lake City, Utah, USA; College of Science, University of Utah, Salt Lake City, Utah, USA; College of Science, University of Utah, Salt Lake City, Utah, USA; College of Science, University of Utah, Salt Lake City, Utah, USA; Columbia College, Department of Biological Sciences, Columbia University, New York, New York, USA; Columbia College, Department of Biological Sciences, Columbia University, New York, New York, USA; College of Science and Technology, Temple University, Philadelphia, Pennsylvania, USA; Armour College of Engineering, Department of Biomedical Engineering, Illinois Institute of Technology, Chicago, Illinois, USA; Department of Pediatrics, University of Utah Spencer Fox Eccles School of Medicine, Salt Lake City, Utah, USA; Department of Pediatrics, University of Utah Spencer Fox Eccles School of Medicine, Salt Lake City, Utah, USA

**Keywords:** career development, medical education, outreach, recruitment

## Abstract

**Background:**

Only 74% of infectious diseases (ID) training positions were filled in the 2022 fellowship match, indicating a need to find increasingly novel and creative solutions for ID recruitment and outreach.

**Methods:**

The websites of 2321 universities and 181 medical schools across the United States were manually searched for the presence of undergraduate clubs and interest groups, respectively, for multiple medical specialties and subspecialties, including ID. Geographic data were used to compare the proximity of ID fellowships to undergraduate institutions.

**Results:**

ID student groups were extremely rare among the categories studied throughout undergraduate institutions (6 out of 2048, or 0.29%). Only 58 of 163 (35.6%) medical schools nationwide included an ID student group. Geographic comparison found that every adult ID fellowship is in the same county and/or city as at least 1 undergraduate institution and 28.5% of adult ID fellowships are in the same zip code as at least 1 undergraduate institution.

**Conclusions:**

The relative paucity of ID student interest groups presents an opportunity for the ID community to begin outreach and recruitment at the undergraduate and medical student levels, specifically through student groups.

The need for infectious diseases physicians continues to grow across the United States. The 2019 Infectious Diseases Society of America (IDSA) Strategic Plan has underscored the need to build a more robust workforce in this subspecialty [1, 2]. However, the 2022 fellowship match highlighted the recent problem of insufficient trainee interest in the field—74% of ID training positions were filled, an 8% decrease from 2021 [3]. Hypothesized reasons for interest in ID training include positive medical education and mentorship experiences, although others have cited the negative effects of relative undercompensation, lack of procedures, and a shift to hospitalist models [4–7]. Various authors have proposed strategies to address this, including changes in medical school and residency curricula, involving medical students and residents in the IDSA, and improving mentorship programs [7]. To meet current and future workforce needs, further innovative solutions must be considered.

Undergraduate and medical school student groups provide one possible avenue for recruitment efforts. Bonura et al. found that ID fellowship applicants often developed an interest in the field before residency and were influenced by mentors and participation in scholarship [4]. Student groups and clubs often have faculty advisors, fundraise to attend national meetings, and offer spaces for collaboration. Consequently, they can be effective in generating interest in ID. However, such groups are uncommon, especially at the undergraduate level. Here, we characterize the prevalence of ID-related student clubs at the undergraduate and medical student levels.

## METHODS

### Presence of Student Groups Nationwide

**Figure 1.**
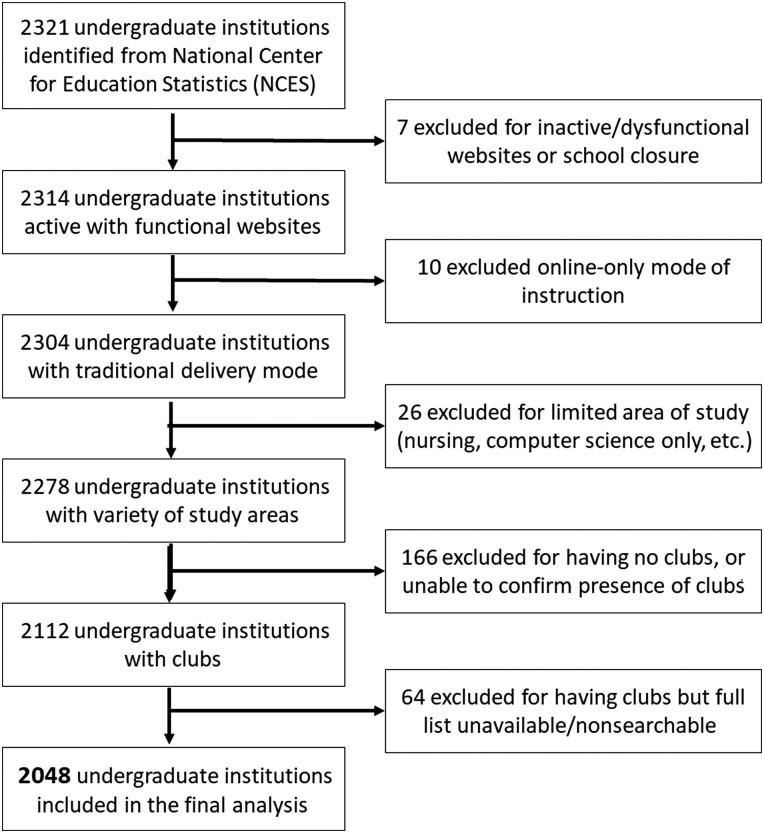
Inclusion criteria for undergraduate institutions evaluated.

In December of 2022, a list was generated of 2321 US institutions with undergraduate enrollments of at least 1000 students from the US Department of Education National Center for Education Statistics (NCES) *College Navigator* system (Figure 1).

Schools were excluded if they were online-only or had a limited study area (ie, computer science–only colleges). From December 2022 to June 2023, investigators searched institutional websites for information on available student clubs and organizations. Schools with no student groups or for which complete lists could not be located were excluded. Each campus was considered a separate institution for multicampus institutions with student groups at multiple campuses; otherwise, all campuses were considered 1 institution. A total of 2048 institutions nationwide met all inclusion criteria.

From the eligible institutions’ websites, we tallied the presence of student groups for science, technology, engineering, and math (STEM), pre-health, emergency medicine, neuroscience/psychology, global/public health, microbiology, and ID. Clubs catering to pre-med, pre–physician assistant, pre-dental, and pre-nursing students were included as pre-health. Paramedic/EMT clubs were included in emergency medicine. Chapters of the Psi Chi Psychology Honors Society were included, while campus mental health clubs were excluded for neuroscience/psychology. Public health clubs were included in a combined group of global/public health. Microbiome clubs were included in the microbiology category. Fungi/mycology clubs were excluded, as a search and review of their websites revealed that their focus was overwhelmingly on foraging and agricultural applications. Immunology clubs were included in an additional search. Only clubs that explicitly mentioned “infectious diseases” in the club title or as a focal point in club descriptions were included for ID. For institutions with multiple levels of study (ie, undergraduate, graduate, professional), student groups were verified for being open to undergraduate participation or were excluded. Descriptive statistics were then applied to better characterize these data.

The authors additionally identified 181 US medical schools with full accreditation by the Liaison Committee on Medical Education (LCME) and American Osteopathic Association (AOA) in July 2023. Authors searched institutional websites for information on available student interest groups. Schools with no interest groups or for which complete lists could not be located were excluded. From the website review, interest groups were tallied for internal medicine, pediatrics, surgery, neurology, psychiatry, obstetrics/gynecology, and emergency medicine, as well as additional internal medicine subspecialty fields like cardiology, rheumatology, nephrology, endocrinology, gastroenterology, and hematology/oncology. Interest groups for public health/preventative medicine and global health were also tallied. Beyond internal medicine subspecialties and core clerkship specialties, further medical specialty interest groups ranging from anesthesiology to wilderness medicine were examined. Likewise, descriptive statistics were then applied to characterize these data.

### Geographic Analysis of Fellowships

The IDSA website was accessed in December of 2022 to construct a list of all adult ID fellowships (n = 165) in the United States and their complete mailing addresses. The ID fellowship zip code and county were matched to the zip code and county of the 2048 undergraduate institutions. Matches were tallied when a fellowship's zip code or county matched an undergraduate institution's zip code.

## RESULTS

### Presence of Student Groups Nationwide

**Figure 2.**
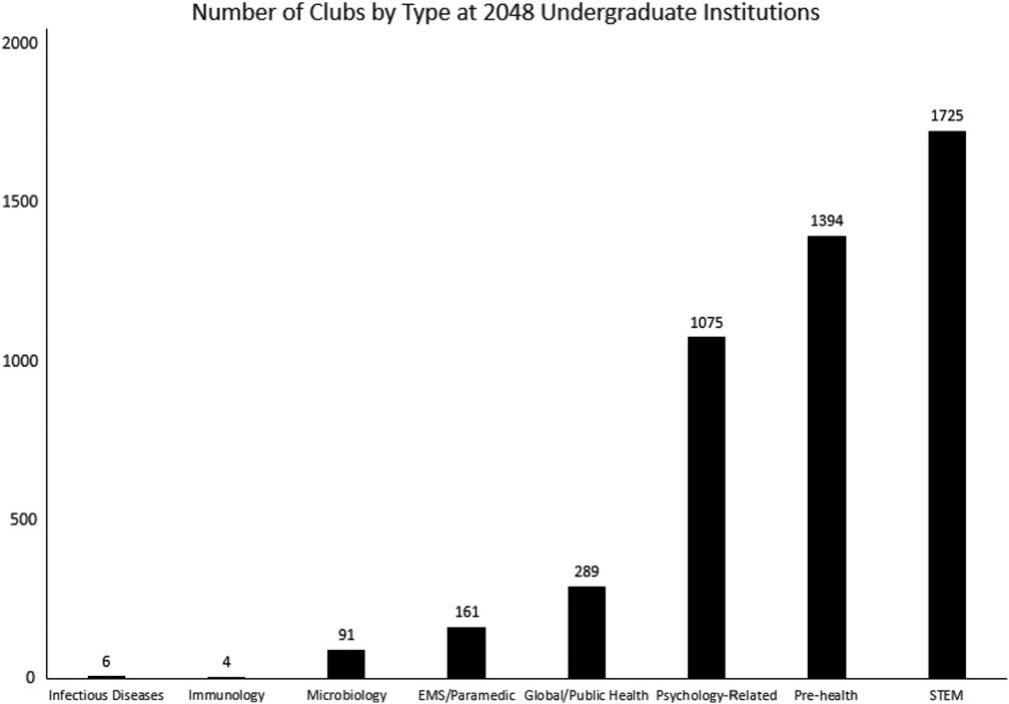
Prevalence of student clubs open to undergraduates at 2048 US institutions with a minimum of 1000 enrolled undergraduate students. Abbreviations: EMS, emergency medical services; STEM, science, technology, engineering, and math.

Six ID clubs were identified at the undergraduate level (Figure 2) from 2048 undergraduate institutions (0.29%). Other health-related student groups ranged from 4 to 1725 clubs nationwide. The 6 ID clubs included “InfectED” at the University of Utah, Operation Outbreak at Brigham Young University (BYU), the Association of Public Health Infectious Disease Students at the University of South Carolina–Columbia, the Infectious Disease Institute Trainee Association at the Ohio State University, the Penn Infectious Disease Club at the University of Pennsylvania, and the Infectious Disease Society at Brown University. BYU's club is the only ID club at a university or institution not affiliated with an ID fellowship or school of medicine.

**Figure 3.**
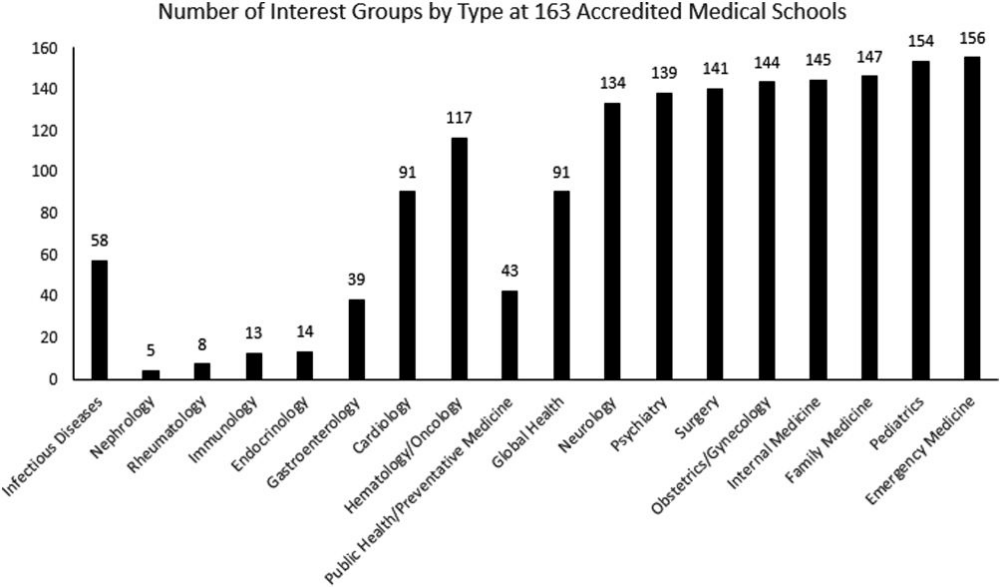
Prevalence of student interest groups at 163 accredited US medical schools.

From the 163 eligible medical schools, 58 ID interest groups were identified (35.6%) (Figure 3; Supplementary Data). Other internal medicine subspecialty groups had a broad range of interest group support: A higher prevalence of interest groups was found for cardiology (n = 91, 55.8%) and hematology/oncology (n = 117, 71.8%). A lower relative prevalence of interest groups was found for gastroenterology (n = 39, 23.9%), endocrinology (n = 14, 8.6%), immunology (n = 13, 8.0%), rheumatology (n = 8, 4.9%), and nephrology (n = 5, 3.1%). Among specialties representing core clerkships in medical school curricula, interest group prevalence was much higher than that of infectious diseases; these included emergency medicine (n = 156, 95.7%), pediatrics (n = 154, 94.5%), family medicine (n = 14, 90.2%), internal medicine (n = 145, 88.9%), obstetrics/gynecology (n = 144, 88.3%), surgery (n = 141, 86.5%), psychiatry (n = 139, 85.3%), and neurology (n = 134, 82.2%). Global health (n = 91, 55.8%) had a higher prevalence, while public health (n = 43, 26.4%) had a lower prevalence than infectious diseases.

Among the 37 included osteopathic medical schools, 6 (16.2%) had an ID interest group. Among the 126 included allopathic medical schools, 52 (41.3%) had an ID interest group.

### Geographic Analysis of Fellowships

Of the 2048 undergraduate institutions, 29.5% (n = 604) are in the same county as an ID fellowship. According to enrollment data from the NCES, this translates to about 5.67 million undergraduate students. Furthermore, another 8 undergraduate institutions do not share the same county but do share the same city as an ID fellowship. Every ID fellowship is either in the same county or city as at least 1 undergraduate institution. At the zip code level, 28.5% of ID fellowships (n = 47) are in the same zip code as at least 1 undergraduate institution, suggesting that nearly a third of academic ID divisions are in very close proximity to an undergraduate student body.

## DISCUSSION

The value of and need for infectious diseases specialists are well recognized [8, 9]. Limited previous research has looked at the role of undergraduate education on specialty choice but has generally focused on undergraduate majors and returned inconclusive or nonsignificant results [10, 11]. Other aspects of undergraduate and medical school education, such as extracurricular activities, deserve consideration as a recruitment opportunity. Previous research on the topic has suggested that enhanced exposure to ID, scholarship opportunities, and role models can increase interest in joining ID [4, 12]. Student clubs and interest groups provide such spaces for students to interact with faculty mentors, attend conferences, and engage in ID and other scientific activities outside of what is possible in the traditional classroom and research settings.

At 2048 undergraduate institutions, only 6 undergraduate ID clubs (0.29%) were identified. ID student groups represent an extremely low prevalence compared with other science, technology, and biomedical and health-related clubs. Microbiology clubs, while rare (n = 91, 4.44%), were more common than ID. Immunology clubs were rarer than ID clubs (n = 4, 0.20%). Interest in global and public health was more prevalent, with 289 (14.6%) undergraduate clubs.

With regards to comparing medical school interest groups, ID (n = 58, 35.6%) was more prevalent than some internal medicine subspecialty groups, like endocrinology (n = 14, 8.6%), immunology (n = 13, 8.0%), rheumatology (n = 8, 4.9%), and nephrology (n = 5, 3.1%). However, ID was still lower prevalence than specialties like cardiology and hematology/oncology, suggesting there is an opportunity for growth. When compared against interest groups for specialties associated with clerkship rotations, ID was much lower in prevalence (Figure 3). Public health/preventative medicine and global health groups together (n = 134, 82.2%) outnumbered ID groups, but this may represent an opportunity to generate further interest in the subspecialty, given the overlap of these fields.

Results from our geographic analysis offer an avenue for outreach. We used the presence of an adult ID fellowship as a proxy for the presence of an ID division and faculty actively involved in medical education and recruitment. Our results indicate that every ID fellowship is in the same county or city as at least 1 undergraduate institution. These data can encourage fellowship directors and faculty to consider local outreach, whether by encouraging the founding of an ID club or providing instruction or mentorship at an undergraduate level. The country's many pre-health clubs offer another way for faculty to get involved. Such clubs often host guest speakers and workshops through which faculty can inspire interest in their specialty. Nationwide, we found 380 total microbiology and global/public health clubs, which could also allow fellowship faculty to take advantage of student interest in adjacent fields toward highlighting ID.

There are limitations to this study and its methodology. Most notably, some websites may not have been updated and maintained. Others may have been updated during the course of the search, which could introduce a source of error or inconsistency. The lack of a student group on an institution's website does not preclude its existence, and thus this study may underrepresent the true prevalence of these clubs to some degree. The zip code proximity data may underrepresent proximity due to institutions having multiple zip codes. Similarly, institutions and ID fellowships may be in different counties. In addition, the pathway from undergraduate and medical students’ engagement in ID to eventual fellowship remains unclear and requires further study.

Our experience with an undergraduate ID club at the University of Utah, InfectED, may be a replicable model for other institutions. InfectED was started in the University of Utah's Honors College following a yearlong experiential course on the bioethics of pandemics co-taught by 2 authors (T.W.J., W.L.H.). Several motivated students started a registered student organization focused on increasing education on infectious diseases on campus and in the Salt Lake Valley through presentations, games, and other club activities. Nearly 2 years after its founding, InfectED sees consistent participation from about 15 students per semester and employs medical-themed community volunteering, journal clubs, and guest speakers as regular activities. Students have cited the guest speakers and the ability to interact in a relaxed environment with medical professionals as the primary draw. ID faculty making themselves available to students at other institutions can achieve similar results. As this is a new interest group, the trajectory of these students’ careers regarding future specialization and interest remains to be seen.

Faculty wishing to encourage interest groups in ID should first identify motivated students who can champion such efforts among their peers. In our experience, this can be accomplished by meeting students through teaching in undergraduate or medical school settings, volunteering for guest lectures in existing courses, reaching out to existing, content-adjacent student organizations (like pre-health or global health groups), and, most importantly, presenting students with opportunities to get to know infectious diseases specialists through events, shadowing, and mentorship. Undergraduate and medical school student organizations often require the drafting of specific constitutions and delegation of student officer roles, which vary by institution. However, student body governments and administration will often provide step-by-step instructions to faculty and student leaders if needed. Faculty often must serve as formal or informal advisors to such newly drafted groups. To encourage participation, events should be planned on a quarterly or semesterly basis around students’ schedules. Faculty advisors’ presence at such events is invaluable, as students often find medical faculty inaccessible otherwise.

While such groups may provide students with encouragement and support related to interest in infectious diseases, further study must be undertaken to relate such early interactions to future specialty and subspecialty selection.

## Supplementary Material

ofad439_Supplementary_Data

## Supplementary Data

Supplementary materials are available at *Open Forum Infectious Diseases* online. Consisting of data provided by the authors to benefit the reader, the posted materials are not copyedited and are the sole responsibility of the authors, so questions or comments should be addressed to the corresponding author.
