# Supplementary material for: Deeper Pathways for Recruitment Efforts: Identifying Opportunities for Undergraduate and Medical Student Involvement in Infectious Diseases
Source: Open Forum Infect Dis. 2023 Aug 23;10(9):ofad439. doi: 10.1093/ofid/ofad439 (PMC10538255; doi:10.1093/ofid/ofad439)
Supplement: ofad439_Supplementary_Data [file ofad439_supplementary_data.docx]

Supplemental Material

Undergraduate Infectious Diseases (ID) Clubs

| Club Name | Institution | Website |
| --- | --- | --- |
| Association of Public Health Infectious Disease Students | University of South Carolina-Columbia | https://garnetgate.sa.sc.edu/organization/aphids |
| InfectED | University of Utah | https://www.honors.utah.edu/infected/ |
| Infectious Disease Institute Trainee Association | Ohio State University | https://idi.osu.edu/education |
| Infectious Disease Society | Brown University | https://www.brown.edu/academics/college/advising/health-careers/student-groups |
| Operation Outbreak | Brigham Young University (BYU) | https://oo.byu.edu/ |
| Penn Infectious Disease Club | University of Pennsylvania | https://pennclubs.com/club/penn-infectious-disease-club |

Full Tally of Medical Specialty Interest Groups Among 161 United States Medical Schools

| **Specialty Interest Group** | **N** | **% (of total 163 schools)** |
| --- | --- | --- |
| Addiction/Recovery | 36 | 22.36% |
| Anesthesiology | 143 | 87.73% |
| Cardiothoracic Surgery | 37 | 22.98% |
| Cardiology | 91 | 55.83% |
| Critical Care | 21 | 12.88% |
| Dermatology | 140 | 85.89% |
| Emergency Med | 156 | 95.71% |
| Endocrinology | 14 | 8.70% |
| ENT/Otolaryngology | 108 | 65.26% |
| Family Med | 147 | 90.18% |
| Gastroenterology | 39 | 22.93% |
| Geriatrics | 67 | 40.10% |
| Global Health | 91 | 55.83% |
| Global Surgery | 31 | 19.02% |
| Hematology/Oncology | 117 | 71.78% |
| Infectious Diseases | 58 | 35.58% |
| Immunology/Allergy | 13 | 7.98% |
| Integrative/Alternative | 34 | 20.86% |
| Internal Medicine | 145 | 88.96% |
| Interventional Radiology | 68 | 41.72% |
| Medical Genetics | 33 | 20.25% |
| Nephrology | 5 | 3.07% |
| Neurology | 134 | 82.21% |
| Neurosurgery | 90 | 55.22% |
| Obstetrics/Gynecology | 144 | 88.34% |
| Ophthalmology | 117 | 71.78% |
| Orthopedics | 131 | 82.21% |
| Pathology | 97 | 59.51% |
| Pediatrics | 154 | 94.48% |
| Plastic Surgery | 80 | 49.08% |
| PM&R | 113 | 69.33% |
| Psychiatry | 139 | 85.28% |
| Public Health/Preventative | 43 | 26.38% |
| Pulmonology | 7 | 4.29% |
| Radiation Oncology | 28 | 17.18% |
| Radiology | 127 | 77.91% |
| Rheumatology | 8 | 4.91% |
| Surgery | 141 | 86.50% |
| Urology | 89 | 54.60% |
| Vascular Surgery | 55 | 33.74% |
| Wilderness Medicine | 95 | 58.28% |

United States Medical Schools Identified with Infectious Diseases Interest Groups

1. Baylor College of Medicine
2. University of Kansas School of Medicine
3. T.H. Chan School of Medicine at the University of Massachusetts
4. University of Arizona College of Medicine - Phoenix
5. David Geffen School of Medicine at UCLA
6. Stanford University School of Medicine
7. University of California, San Diego School of Medicine
8. Frank H. Netter MD School of Medicine at Quinnipiac University
9. University of Connecticut School of Medicine
10. George Washington University School of Medicine and Health Sciences
11. Georgetown University School of Medicine
12. Florida International University Herbert Wertheim College of Medicine
13. University of Florida College of Medicine
14. University of Miami Leonard M. Miller School of Medicine
15. USF Health Morsani College of Medicine
16. Emory University School of Medicine
17. Loyola University Chicago Stritch School of Medicine
18. Northwestern University Feinberg School of Medicine
19. University of Illinois College of Medicine
20. Indiana University School of Medicine
21. University of Iowa Roy J. and Lucille A. Carver College of Medicine
22. University of Kentucky College of Medicine
23. Johns Hopkins University School of Medicine
24. Uniformed Services University of the Health Sciences, F. Edward Hébert School of Medicine
25. University of Maryland School of Medicine
26. Michigan State University College of Human Medicine
27. Wayne State University School of Medicine
28. University of Minnesota Medical School
29. University of Missouri-Columbia School of Medicine
30. Washington University in St. Louis School of Medicine
31. University of Nebraska College of Medicine
32. Cooper Medical School of Rowan University
33. Rutgers New Jersey Medical School
34. University of New Mexico School of Medicine
35. Donald and Barbara Zucker School of Medicine at Hofstra/Northwell
36. New York Medical College
37. University of Rochester School of Medicine and Dentistry
38. Weill Cornell Medicine
39. University of North Carolina School of Medicine
40. University of Cincinnati College of Medicine
41. Drexel University College of Medicine
42. Raymond and Ruth Perelman School of Medicine at the University of Pennsylvania
43. Sidney Kimmel Medical College at Thomas Jefferson University
44. University of South Carolina School of Medicine, Columbia
45. University of South Carolina School of Medicine, Greenville
46. Texas Tech University Health Sciences Center School of Medicine
47. The University of Texas Health Science Center at San Antonio Joe R. and Teresa Lozano Long School of Medicine
48. University of Texas Southwestern Medical School
49. Spencer Fox Eccles School of Medicine at the University of Utah
50. Virginia Commonwealth University School of Medicine
51. University of Washington School of Medicine
52. University of Wisconsin School of Medicine and Public Health
53. Des Moines University College of Osteopathic Medicine
54. Marian University College of Osteopathic Medicine
55. Pacific Northwest University of Health Sciences College of Osteopathic Medicine
56. Rowan-Virtua School of Osteopathic Medicine
57. Sam Houston State University College of Osteopathic Medicine
58. University of North Texas Health Science Center Texas College of Osteopathic Medicine

Of 58 ID interest groups, 46 are at institutions with linked ID Fellowships. 12 are at institutions with no ID Fellowships. Of the 12, all 6 IDIGs at DO schools are represented, as well as 6 more IDIGs with no ID Fellowship at the institution. There are

1. (MD) Frank H. Netter MD School of Medicine at Quinnipiac University
2. (MD) Donald and Barbara Zucker School of Medicine at Hofstra/Northwell
3. (MD) Drexel University College of Medicine
4. (MD) Uniformed Services University of the Health Sciences, F. Edward Hébert School of Medicine
5. (MD) Florida International University Herbert Wertheim College of Medicine
6. (MD) University of Arizona College of Medicine - Phoenix
7. (DO) Rowan-Virtua School of Osteopathic Medicine
8. (DO) Marian University College of Osteopathic Medicine
9. (DO) Des Moines University College of Osteopathic Medicine
10. (DO) University of North Texas Health Science Center Texas College of Osteopathic Medicine
11. (DO) Sam Houston State University College of Osteopathic Medicine
12. (DO) Pacific Northwest University of Health Sciences College of Osteopathic Medicine
